# Typhlitis induced by *Histomonas meleagridis* affects relative but not the absolute *Escherichia coli* counts and invasion in the gut in turkeys

**DOI:** 10.1186/s13567-021-00962-6

**Published:** 2021-06-22

**Authors:** Mohamed Kamal Abdelhamid, Ivan Rychlik, Claudia Hess, Tamas Hatfaludi, Magdalena Crhanova, Daniela Karasova, Julia Lagler, Dieter Liebhart, Michael Hess, Surya Paudel

**Affiliations:** 1 Clinic for Poultry and Fish Medicine, Department for Farm Animals and Veterinary Public Health, University of Veterinary Medicine, Vienna, Austria; 2 Department of Pathology, Faculty of Veterinary Medicine, Beni-Suef University, Beni-Suef, Egypt; 3 Department of Immunology, Veterinary Research Institute, 62100 Brno, Czech Republic; 4 Christian Doppler Laboratory for Innovative Poultry Vaccines (IPOV), University of Veterinary Medicine, Vienna, Austria; 5 Present Address: Section of Immunology, Vetsuisse Faculty, University of Zurich, Zurich, Switzerland

**Keywords:** *Escherichia coli*, *Histomonas meleagridis*, Turkeys, Caecal microbiota, Inflammation, Penetration

## Abstract

Unlike in chickens, dynamics of the gut microbiome in turkeys is limitedly understood and no data were yet published in context of pathological changes following experimental infection. Thus, the impact of *Histomonas meleagridis*-associated inflammatory changes in the caecal microbiome, especially the *Escherichia coli* population and their caecal wall invasion in turkeys was investigated. Birds experimentally inoculated with attenuated and/or virulent *H. meleagridis* and non-inoculated negative controls were divided based on the severity of macroscopic caecal lesions. The high throughput amplicon sequencing of 16SrRNA showed that the species richness and diversity of microbial community significantly decreased in severely affected caeca. The relative abundances of operational taxonomic units belonging to *Anaerotignum lactatifermentans*, *E. coli*, and *Faecalibacterium prausnitzii* were higher and paralleled with a decreased abundances of those belonging to *Alistipes putredinis*, *Streptococcus* *alactolyticus*, *Lactobacillus salivarius* and *Lactobacillus reuteri* in birds with the highest lesion scores. Although the relative abundance of *E. coli* was higher, the absolute count was not affected by the severity of pathological lesions. Immunohistochemistry showed that *E. coli* was only present in the luminal content of caecum and did not penetrate even severely inflamed and necrotized caecal wall. Overall, it was demonstrated that the fundamental shift in caecal microbiota of turkeys infected with *H. meleagridis* was attributed to the pathology induced by the parasite, which only led to relative but not absolute changes in *E. coli* population. Furthermore, *E. coli* cells did not show tendency to penetrate the caecal tissue even when the intestinal mucosal barriers were severely compromised.

## Introduction

A normal gut microbial community and integrity of the intestinal wall in poultry are essential for nutrient digestion, production of beneficial short chain fatty acids and prevention of disease by competitive exclusion or blocking of pathogen colonization [1, 2]. Any shifts of the resident microbiota might compromise all or some of these functions [3] leading to reduction in intestinal barrier function potentially resulting in bacterial translocation from gut to systemic organs [4, 5]. The knowledge on the composition of complex gut microbiome has enhanced with the application of next generation sequencing [6]. Applying this technique, intestinal microbiota and their interactions with the chicken host have been elucidated in experimental infection models with several pathogens, for instance, infection with *Eimeria tenella* led to relative increase of *Enterobacteriaceae* and decrease of *Lactobacillaceae* [7–10]. Likewise, *Histomonas meleagridis* infection resulted in reduced gut microbial richness and diversity favouring caecal colonization of avian pathogenic *Escherichia coli* in chicken layers [4]. In turkeys, such studies are still limited and dynamics of enteric microbiota, especially in the presence of inflammation and necrosis remains to be understood. Previous studies in turkeys focused on characterization of the microbiota along the gastrointestinal tract or decipher changes induced by antibiotic treatment, different litter management or natural infection with haemorrhagic enteritis virus [11–14]. Additional studies associated composition of turkey gut microbiota with production of short chain fatty acids in the gut or increase in the body weight [15–17].

*Escherichia coli* is a Gram-negative bacterium, a member of the *Enterobacteriaceae* and resides in the lower digestive tract of chickens and turkeys where it colonizes in the first 24 h post-hatching [18, 19]. Avian colibacillosis caused by *E. coli* is reported to be responsible for fibrinous peritonitis, hepatitis, polyserositis in turkeys and causing economic losses [20, 21]. Furthermore, some of the *E. coli* isolates from clinical cases of turkey cellulitis were characterized to be avian pathogenic *E. coli* based on the presence of virulence associated genes and phylogenetic groups [22]. Although the demarcations between pathotypes are not very clear, some of the intestinal commensals might carry virulence associated genes and the isolates could show opportunistic or potentially pathogenic nature in birds [23]. In contrast to chickens, experimental and field studies related to *E. coli* infection in turkeys are very scarce. The majority of experimental studies in turkeys involved respiratory routes of infection and were focused to elucidate the impact of stress factors or other co-residing pathogens in the progression of *E. coli* infection [24–27]. Thus, the relationship between *E. coli* populations residing in the gut and systemic bacteremia in turkeys is largely unknown.

*Histomonas meleagridis*, a protozoal parasite is very well known to cause severe inflammation and necrosis in caeca and liver of experimentally infected turkeys, and in vitro mutual interaction of *E. coli* with the parasite has been shown previously [28]. In addition, in a previous co-infected model in chicken layers, *H. meleagridis* enhanced the penetration of *E. coli* throughout the caecal tissue [4]. The present study was therefore conducted in turkeys with the following two main objectives: (i) to investigate the dynamics of intestinal *E. coli* population and a possible microbial shift related to the pathology induced by *H. meleagridis* infection in the gut, and (ii) to assess the consequences of inflammation and necrosis on caecal wall penetration with *E. coli* and their systemic translocation.

## Materials and methods

### Birds and housing

Thirty-three one-day-old commercial Hybrid Converter turkeys (Hendrix Genetics, Boxmeer, the Netherlands) were randomly divided into four groups (Table 1). Each group of birds was placed in a separate negatively pressured room on deep litter.

**Table 1:** **Design of the animal experiment**

Group	Experimental inoculation (days of life)		Necropsy and sampling (days of life)		
	1	28	35	42	49
Negative control (*n* = 9)	*E. coli* DH5α	*E.* *coli* DH5α	3^a^	3	3
Vaccinated (*n* = 9)	No	Attenuated *H.* *meleagridis* co-cultivated with *E.* *coli* DH5α	3	3	3
Infected (*n* = 6)	No	Virulent *H.* *meleagridis* co-cultivated with *E. coli* DH5α	3	3	NA^b^
Vaccinated + infected (*n* = 9)	Attenuated *H. meleagridis* co-cultivated with *E. coli* DH5α	Virulent *H.* *meleagridis* co-cultivated with *E.* *coli* DH5α	2^c^	3	3

^a^Number of necropsied birds at each time point.

^b^Not applicable.

^c^One bird showed swollen hock joints and was euthanized prior to the necropsy date.

### Preparation of *H. meleagridis* cultures for inoculation

The virulent (in vitro passage of 28 times) or attenuated (in vitro passage of 301 or 302 times) clonal culture of *H. meleagridis*/Turkey/Austria/2922-C6/04 co-cultivated with the bacterial strain *E. coli* DH5α, as a supplement for propagation of the parasite [28] was used for inoculation. The inocula medium consisted of Medium 199 with Earle’s salts, l-glutamine, 25 mM HEPES and l-amino acids (Gibco™ Invitrogen, Austria), 15% fetal calf serum (Gibco™ Invitrogen) and rice starch, 0.25% (w/v) (Sigma-Aldrich, Vienna, Austria). The number of viable attenuated and virulent *H. meleagridis* cells was determined using trypan blue stain and a Neubauer hemocytometer (Sigma-Aldrich, St. Louis, MO, USA) to adjust the appropriate number in each inoculum. For *E. coli* DH5α, colony-forming units (CFU) were counted from serial dilutions of the inoculum on coliform agar plates after incubation at 37 °C for 24 h.

### Experimental design

As illustrated in Table 1, different groups of birds were treated as follows: control birds (*n* = 9) were inoculated with culture media containing only 7.2 × 10^6^ CFU and 1 × 10^7^ CFU of *E. coli* DH5α at 1 and 28 days of life, respectively; vaccinated birds (*n* = 9) were inoculated with 6 × 10^5^ cells of attenuated *H. meleagridis* (passage 301) co-cultured with 1.2 × 10^8^ CFU of *E. coli* DH5α at 28 days of life; infected birds (*n* = 6) were inoculated with 6 × 10^5^ cells of virulent *H. meleagridis* (passage 28) co-cultured with 9.9 × 10^7^ CFU of *E. coli* DH5α at 28 days of life; vaccinated + infected birds (*n* = 9) were inoculated with 6 × 10^5^ cells of attenuated *H. meleagridis* (passage 302) co-cultured with 1.2 × 10^8^ CFU of *E. coli* DH5α and 6 × 10^5^ cells of virulent *H. meleagridis* (passage 28) co-cultured with 9.9 × 10^7^ CFU of *E. coli* DH5α at 1 and 28 days of life, respectively. All inocula were administered via oral and cloacal routes, and the procedures were followed as described previously [29]. The birds were examined daily for clinical signs. Necropsy and sampling were performed in three birds from each group at 7 and 14 days post *H. meleagridis* infection (dpi), with an exception at 7 dpi when only two birds were killed from the vaccinated + infected birds. Finally, at 21 dpi, the remaining three birds from each group were killed and sampled, except from the infected group where no birds were left for necropsy.

### Macroscopic lesions in caeca

During necropsy, macroscopic lesions in caeca were recorded and lesion scores (LSs) were assigned based on the previously published scoring scheme ranging from 0 to 4 [30].

### Microscopic examination

Caecal tissues from all birds were fixed in 10% neutral buffered formalin, dehydrated, embedded in paraffin, sectioned at 5 µm thickness and stained with hematoxylin and eosin.

### Bacteriology

For the quantification of bacterial load of *E. coli* in caecum, liver and spleen, samples were collected from all birds during necropsy, homogenized and tenfold serial dilution suspensions in phosphate-buffered saline (PBS) were plated on MacConkey agar (Neogen, Heywood, UK) plates in duplicates. Following incubation of plates at 37 °C for 24 h, *E. coli* CFUs were counted and the bacterial loads were calculated as CFU/g of respective organs.

### Microbiota analysis

Caecal contents from each bird were collected in sterile 1.5 mL tubes during necropsy. In case lesions were confined to one side, caecal content of the affected caeca was collected. All samples were stored at −80 °C until further processing. Caecal content was homogenized in a MagNALyzer (Roche). Following homogenization, the DNA was extracted using a QIAamp DNA Stool Mini Kit according to the manufacturer’s instructions (Qiagen, Germany). The DNA concentration was determined with a spectrophotometer and samples diluted to 5 ng/mL were used as a template in PCR with forward primer 5′-*TCGTCGGCAGCGTCAGATGTGTATAAGAGACAG*-MID-GT-CCTACGGGNGGCWGCAG-3′ and reverse primer 5′-*GTCTCGTGGGCTCGGAGATGTGTATAAGAGACAG*-MID-GT-GACTACHVGGGTATCTAATCC-3′. The sequences in italics served for index ligation whereas the underlined sequences allowed for amplification over the V3/V4 region of 16S rRNA genes. MIDs represent different sequences of 5, 6, 7, or 9 base pairs in length which were used to identify individual samples after the whole sequencing run. PCR amplification was performed using a KAPA HiFi Hot Start Ready Mix kit (Kapa Biosystems) and the resulting PCR products were purified using AMPure beads. In the next step, the PCR product concentration was determined with a spectrophotometer before the DNA was diluted to 100 ng/μL and groups of 14 PCR products with different MID sequences were indexed with the same index from Nextera XT Index Kit following the manufacturer’s instructions (Illumina). The next set of 14 PCR products with different MID sequences were indexed with the next index from the Nextera XT Index kit thus allowing an increase in the number of samples analyzed in a single sequencing run. Prior to sequencing, the concentration of differently indexed samples was determined using a KAPA Library Quantification Complete kit (Kapa Biosystems). All indexed samples were diluted to 4 ng/μL and 20 pM phiX DNA was added to a final concentration of 5% (v/v). Sequencing was performed using MiSeq Reagent Kit v3 (600 cycle) and MiSeq apparatus according to the manufacturer’s instructions (Illumina). Quality trimming of the raw reads was performed using TrimmomaticPE v0.32 with sliding window 4 bp and quality read score equal or higher than 20 [31]. Minimal read length must have been at least 150 bp. The fastq files generated after quality trimming were uploaded into QIIME software [32]. Forward and reverse sequences were joined and in the next step chimeric sequences were predicted and excluded by the slayer algorithm. The resulting sequences were then classified by RDP Seqmatch with an OTU (operational taxonomic unit) discrimination level set to 97%.

### Immunohistochemistry

In order to investigate the colonization and penetration of caecal tissues by *E. coli*, paraffin embedded samples of caeca from all birds were processed for immunohistochemistry. Further, to validate the findings from the actual study, caeca of 14 turkeys from infected groups with monoxenic (*n* = 8) or xenic (*n* = 6) cultures of *H. meleagridis* and control birds (*n* = 4) from previously published experiments were included [29, 33]. Likewise, caecal samples of naturally infected turkeys showing severe fibrinous typhlitis from two field cases were also considered. The IHC protocol for the detection of *E. coli* was followed as described previously [4]. Briefly, tissue sections mounted on charged glass slides were dewaxed, rehydrated and incubated overnight with a primary monoclonal antibody (anti-*E. coli* LPS antibody (2D7/1), ab35654, Abcam, Austria). Following incubation, slides were washed with PBS and biotinylated anti-mouse IgG antibody (Vector Laboratories, Austria) was added. Then the vectastain ABC Kit and DAB substrate kit (Vector Laboratories) were used for visualizing the bound antibody. Finally, the sections were counter-stained with Mayer's haematoxylin (Merck KGaA, Austria) and observed under a microscope.

### Statistical analysis

Data of alpha diversity matrices and *E. coli* count were analyzed by one-way ANOVA followed by Tukey’s multiple comparison post hoc test. The Spearman’s rank correlation coefficient (r) was used to evaluate relationship between caecal LSs and *E. coli* count in the caecum. All analyses were performed in SPSS (IBM^®^ SPSS^®^ version 25; IBM cooperation, New York, USA). The *p* value < 0.05 was considered as statistically significant.

## Results

### Clinical signs

One bird from the vaccinated + infected group showed swollen hock joints and was euthanized before scheduled killing. No clinical signs were seen in rest of birds.

### Macroscopic lesions

**Figure 1:**
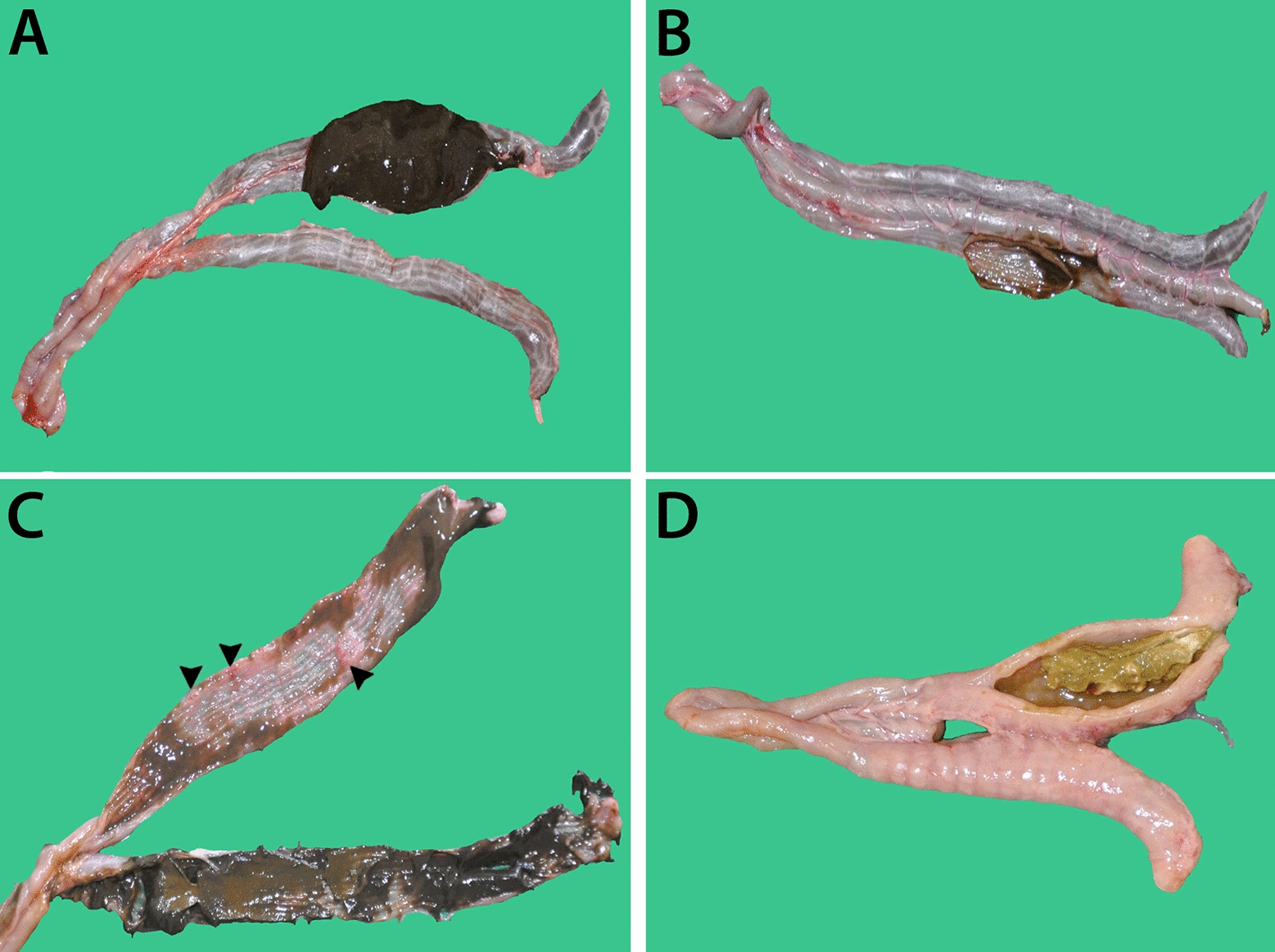
**Macroscopic lesions in caecum of turkeys in different groups.** **A** Negative control group, not inoculated with *H. meleagridis*; **B** no lesion group, inoculated with* H. meleagridis;* **C** mild lesion group, inoculated with *H. meleagridis* (hemorrhages are shown with arrow heads); **D** severe lesion group, inoculated with *H. meleagridis*.

The early-euthanized bird with swollen hock joints showed arthritis. The control birds that were not inoculated with *H. meleagridis* were devoid of pathological lesions similar to those which received attenuated histomonads (LS 0, Figure 1A). Following inoculation of birds with attenuated and/or virulent *H. meleagridis* strains, severity of caecal lesions varied, and LS 0, 1, 3 and 4 were recorded (Figures 1B–D). Therefore, birds were categorized into four groups according to their inoculation status and severity of caecal lesions for the analysis of sequencing, bacteriology and IHC data (Table 2). These groups were as follows: (i) negative control (*n* = 9; birds not inoculated with *H. meleagridis*, LS 0), (ii) no lesion (*n* = 11; birds inoculated with *H. meleagridis* that showed LS 0), (iii) mild lesion (*n* = 6; birds inoculated with *H. meleagridis* that showed LS 1), and (iv) severe lesion (*n* = 6; birds inoculated with *H. meleagridis* that showed LS 3 or 4). Negative control birds were separated from *H. meleagridis* inoculated birds with no lesion to observe possible differences induced by parasite inoculation. The presence of compact fibrinous mass in the lumen of one or both caeca are allocated in the macroscopic LS 3 or LS 4 [30]. As the samples were always taken from the affected caecum, birds showing LS 3 and 4 were grouped together.

**Table 2:** **Number of turkeys in different groups based on the severity of macroscopic lesions in caecum for the analysis of microbiota, E. coli count and immunohistochemistry data**

Treatment	Group			
	Negative control (*n* = 9)	No lesion (*n* = 11)	Mild lesion (*n* = 6)	Severe lesion (*n* = 6)
Negative control	9	0	0	0
Vaccinated	0	9	0	0
Infected	0	0	3	3
Vaccinated + infected^a^	0	2	3	3

^a^One bird was euthanized prior to the scheduled necropsy date, thus is excluded from the total of this group.

### Histopathology

**Figure 2:**
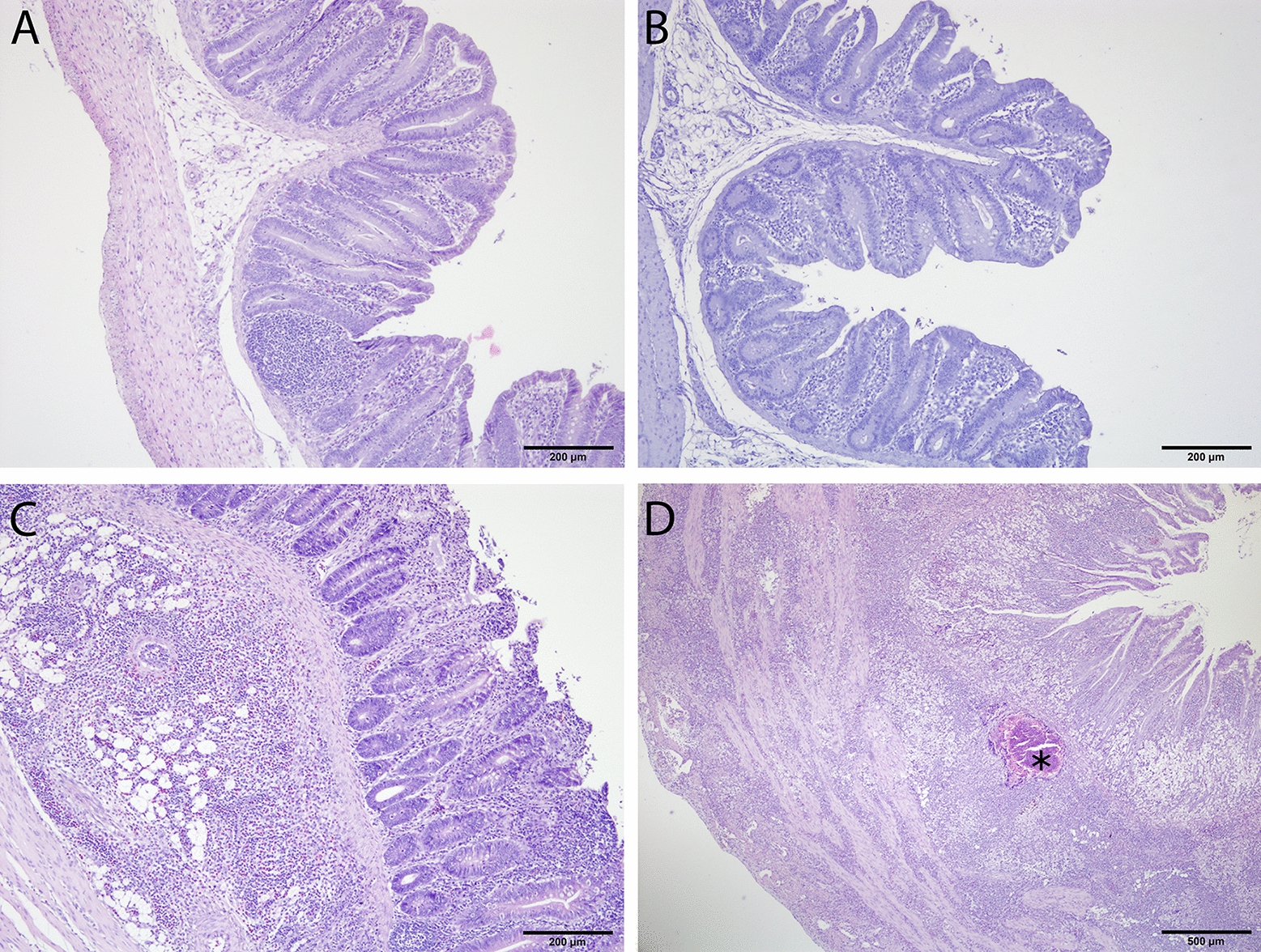
**Histopathology in caecum of turkeys in different groups.** **A** Negative control group, not inoculated with *H. meleagridis,* tissues are normal*;* **B** no lesion group, inoculated with *H. meleagridis*, tissues are normal; **C** mild lesion group, inoculated with *H. meleagridis,* erosion of mucosal epithelium with heterophilic/eosinophilic cells infiltration extended to submucosa; **D** severe lesion group, inoculated with *H. meleagridis*, extensive necrosis with pyogranulomatous reaction (asterisk) and transmural infiltration of inflammatory cells.

No histological lesions were found in caeca of birds from negative control and no lesion groups (Figures 2A and B). Birds belonging to the mild lesion group showed mucosal erosion with heterophilic/eosinophilic infiltration limited to lamina propria and submucosa (Figure 2C). Severe necrosis in mucosa with inflammatory cells extended transmurally was observed in birds assigned to the severe lesion group (Figure 2D).

### Bacteriology

**Figure 3:**
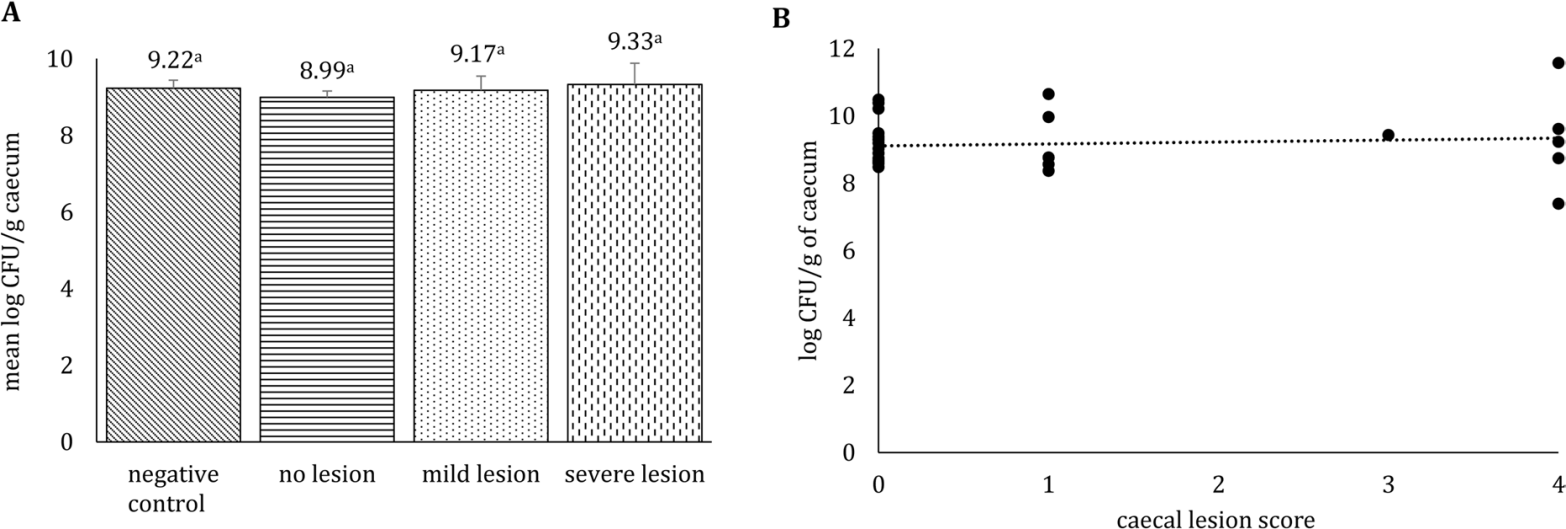
**Average *E. coli* counts (A) and their correlation with the severity of macroscopic lesions (B) in caeca of turkeys.** **A** Groups are as follows: negative control (birds not inoculated with *H. meleagridis*), no lesion (birds inoculated with *H. meleagridis* that showed no lesions), mild lesion (birds inoculated with *H. meleagridis* that showed LS 1), severe lesion (birds inoculated with *H. meleagridis* that showed LS 3 or 4). Results are expressed as mean±SEM, values with different letters are statistically significant; **B** Spearman’s correlation coefficient of caecal lesion score with bacterial load of *E. coli* count in caeca. Lesion scores were assigned based on the previously published scoring scheme [31].

Pure colonies of *Streptococcus* sp. were isolated from the affected joint of the early euthanized bird. Average bacterial counts of *E. coli* in caeca of birds from different groups ranged from 8 to 9 log CFU/g and differences among groups were not statistically significant (Figure 3A). Although a very slight tendency of positive correlation was observed between the severity of caecal macroscopic LSs and the *E. coli* load but this was not statistically significant (r = 0.11; Figure 3B). In systemic organs, one bird from the negative control group was positive in spleen and contained 1.56 log CFU/g of *E. coli*. In another bird from the no lesion group that was inoculated with attenuated *H. meleagridis*, liver and spleen were found positive with 3.1 log CFU/g and 3 log CFU/g of *E. coli*, respectively.

### Caecal microbiota

**Figure 4:**
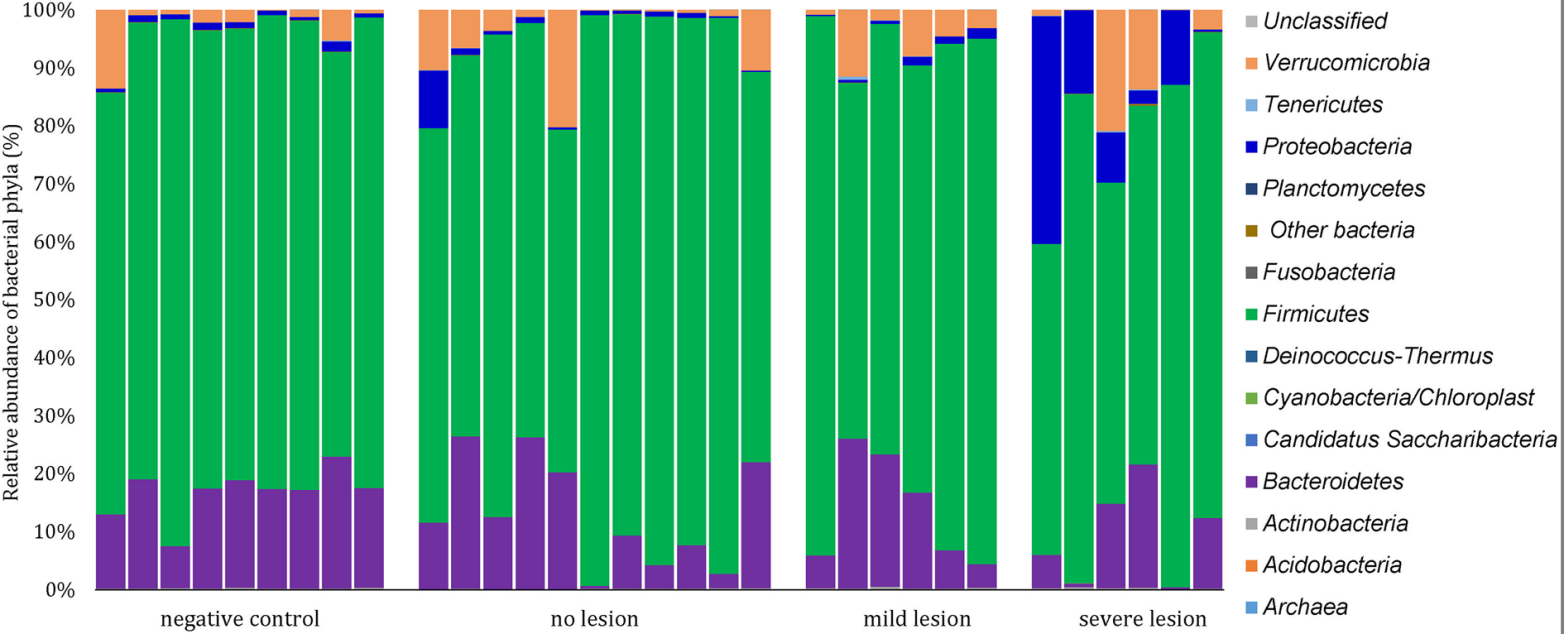
**Relative abundance (%) of OTUs representing the caecal microbiota at phylum level in turkeys.** Groups are as follows: negative control (birds not inoculated with *H. meleagridis*), no lesion (birds inoculated with *H. meleagridis* that showed no lesions), mild lesion (birds inoculated *H. meleagridis* that showed LS 1), severe lesion (birds inoculated with *H. meleagridis* that showed LS 3 or 4).

The OTUs identified in caecal samples were classified into 13 bacterial phyla and one archaeal phylum. Overall *Firmicutes* (78.2%), *Bacteroidetes* (13.3%), *Acidobacteria* (7.4%), *Verrucomicrobia* (4.8%) and *Proteobacteria* (3.4%) were the most dominant phyla (Figure 4).

**Figure 5:**
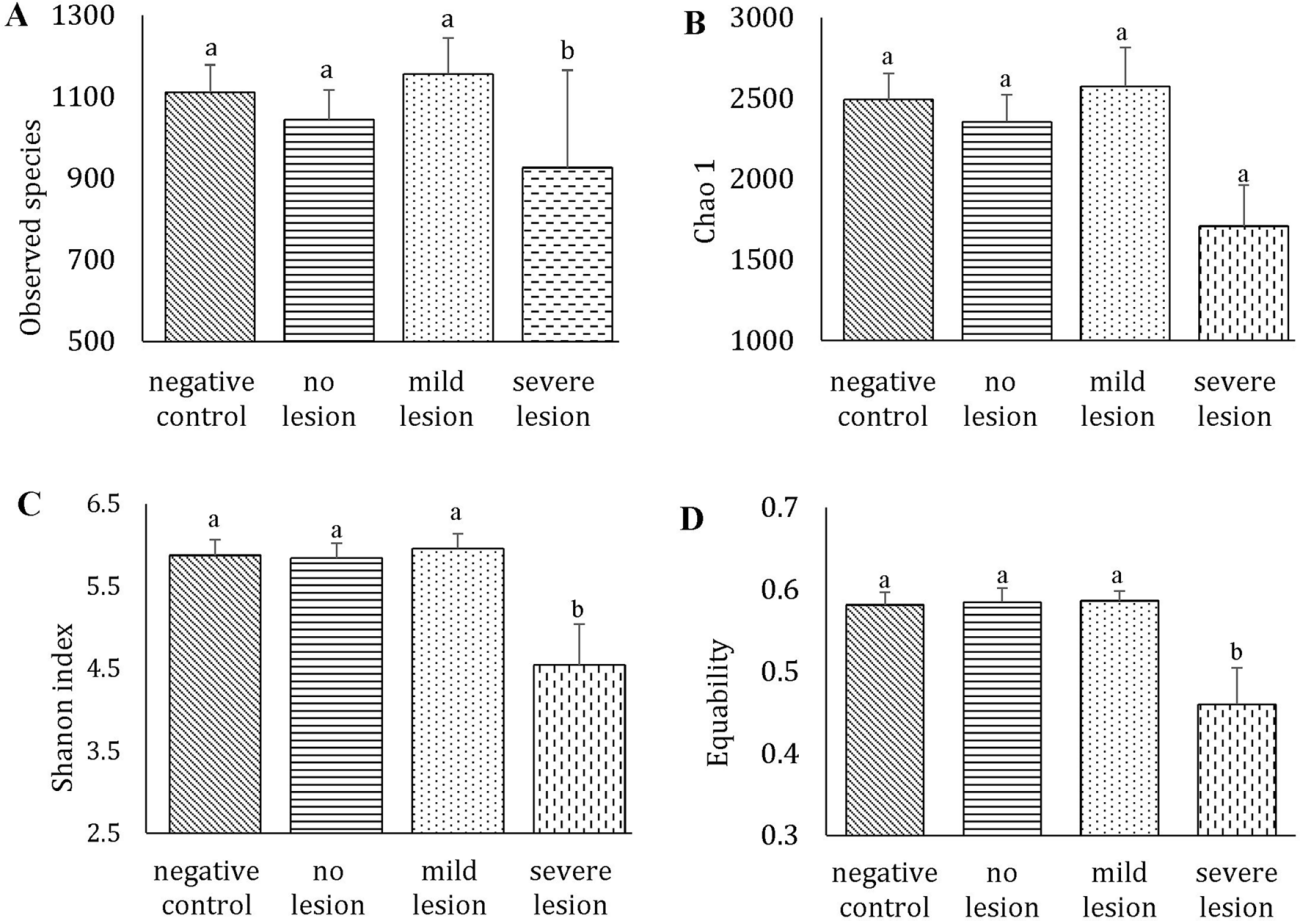
**Alpha diversity indices of microbial community in caeca of turkeys.** **A** Observed species, estimates the total number of OTUs; **B** Chao1, estimates the OTUs richness; **C** shannon index, an estimation of microbial diversity; **D** equability, estimates OTUs evenness. Groups are as follows: negative control (birds not inoculated with *H. meleagridis*), no lesion (birds inoculated with *H. meleagridis* that showed no lesions), mild lesion (birds inoculated with *H. meleagridis* that showed LS 1), severe lesion (birds inoculated with *H. meleagridis* that showed LS 3 or 4). Results are shown as mean ± SEM. Different letters denote statistically significant differences.

Bacterial diversity and richness was evaluated among the four groups (negative control, no lesion, mild lesion and severe lesion) using alpha diversity indices (Observed species, Chao 1, Shannon index and Equability). Bacterial diversity and richness significantly decreased in birds of the severe lesion group compared to others (Figures 5A–D).

**Figure 6:**
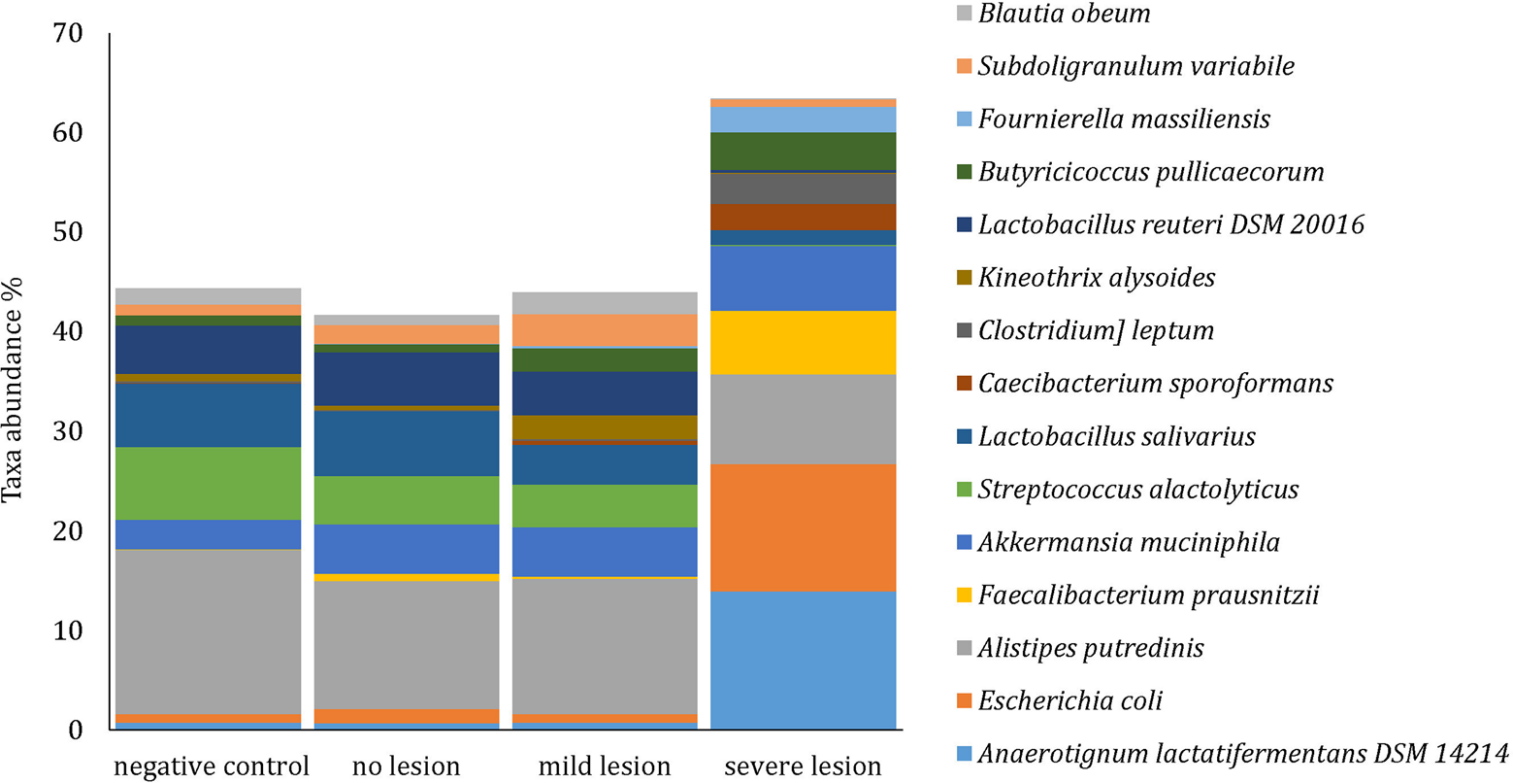
**Mean relative abundance (%) of the 15 most abundant bacterial species in caeca of turkeys.** Groups are as follows: negative control (birds not inoculated with *H. meleagridis*), no lesion (birds inoculated with *H. meleagridis* that showed no lesions), mild lesion (birds inoculated with *H. meleagridis* that showed LS 1), severe lesion (birds inoculated with *H. meleagridis* that showed LS 3 or 4).

The relative abundance of OTUs was analysed at species level and, where possible, confirmation of species was done by aligning with available sequences using nucleotide BLAST or RNA/ITS databases. Out of 15 most abundant bacterial species, the relative abundance of *Anaerotignum lactatifermentans*, *E. coli*, *Faecalibacterium prausnitzii*, *Akkermansia muciniphila*, *Caecibacterium sporoformans*, *Clostridium leptum* and *Butyricicoccus pullicaecorum* were more abundant in birds of the severe lesion group in comparison to other groups. In contrary, *Alistipes putredinis*, *Streptococcus alactolyticus*, *Lactobacillus salivarius*, *Lactobacillus reuteri* were less abundant in birds with the highest lesion scores (Figure 6).

### Immunohistochemistry

**Figure 7:**
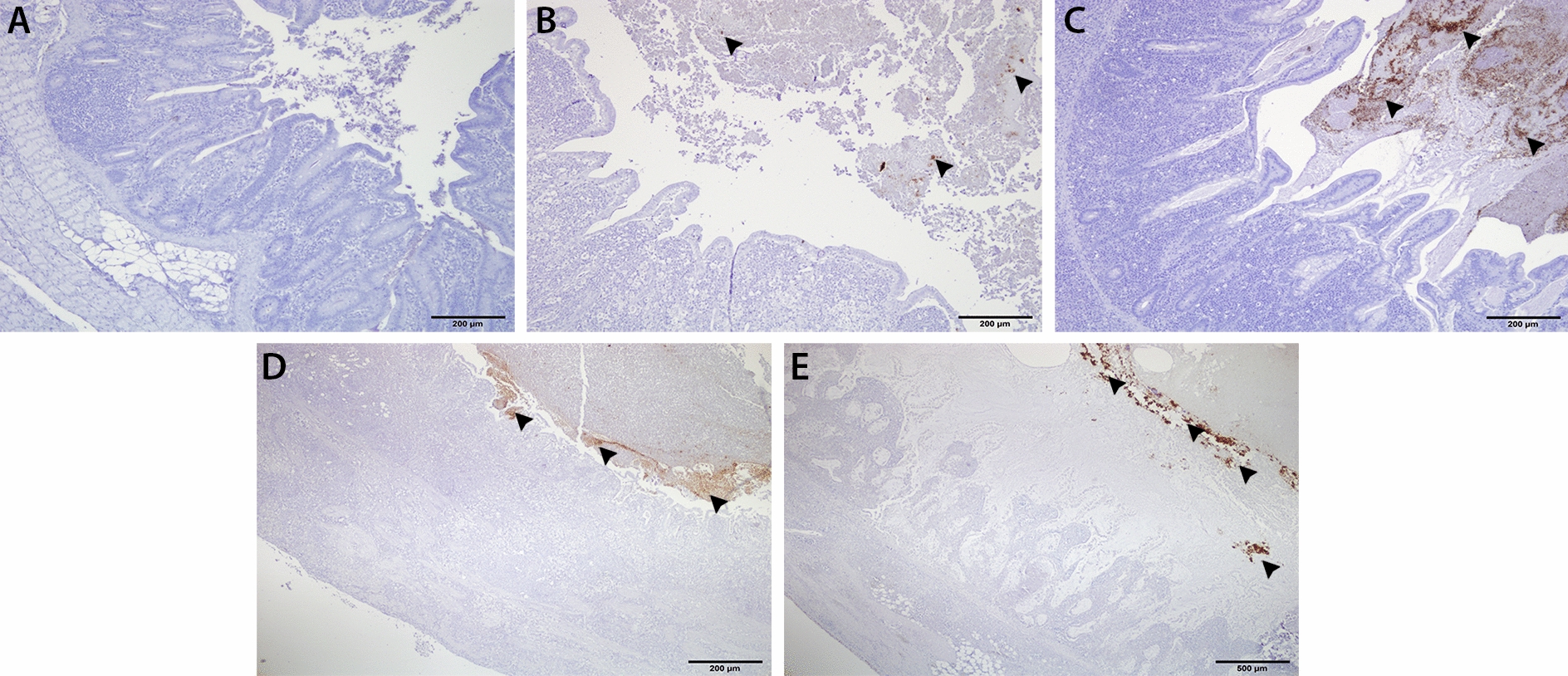
**Immunohistochemical detection of *E. coli* in the caecal samples of turkeys.** **A** Negative control, absence of immunostaining for *E. coli*; **B** presence of *E. coli* in the caecal content (arrow heads) of a sample from the severe lesion group that was infected with *H. meleagridis*; **C**–**E** profound presence of *E. coli* (arrow heads) in the caecal content without any infiltration into the caecal wall, caeca are severely inflamed and necrotized after experimental infection with monoxenic *H. meleagridis* culture (**C**), xenic *H. meleagridis* culture (**D**) natural infection from a field case **E**.

*E. coli* cells were detected in caecal luminal content of one and three birds from the mild lesion and the severe lesion groups, respectively (Table 3). *E. coli* did not penetrate into the caecal wall of birds from any of the groups (Figures 7A and B). In samples taken from previously published experimental studies [29, 33], *E. coli* cells were detected in the luminal content of 3 out of 8 turkeys inoculated with monoxenic *H. meleagri*dis culture (Figure 7C) and in 4 out of 6 turkeys inoculated with xenic *H. meleagri*dis culture, in which some *E. coli* cells were attached on the necrotic epithelium of the caecal mucosa (Figure 7D). None of the samples from both trials showed infiltration of *E. coli* in the caecal wall. Likewise, caecal content and necrotic epithelium of mucosal surface were positive for *E. coli* while layers of caecal wall were negative in samples taken from field cases of histomonosis in turkeys (Figure 7E).

**Table 3:** **Detection of** ***E. coli*** **with immunohistochemistry in the caecum ﻿of turkeys**

Sample type	Caecal lumen (content)	Caecal wall
Infected with monoxenic *H. meleagridis* (actual trial)^a^		
Negative control (*n* = 9)	–^b^	–
No lesion (*n* = 11)	–	–
Mild lesion (*n* = 6)	1^c^	–
Severe lesion (*n* = 6)	3	–
Infected with monoxenic *H. meleagridis* [29]		
Negative control (*n* = 2)	–	–
LS 2 (*n* = 1)	–	–
LS 3 (*n* = 3)	2	–
LS 4 (*n* = 4)	1	–
Infected with xenic *H. meleagridis* [33]		
Negative control (*n* = 2)	–	–
LS 3 (*n* = 1)	1	–
LS 4 (*n* = 5)	3 (caecal content and/or necrotic mucosal epithelium)	–
Field cases with histomonosis		
* n* = 2	2 (caecal content and necrotic mucosal epithelium)	–

^a^For group allocation, refer to Table2.

^b^Negative.

^c^Number of positive samples.

## Discussion

The gut is the reservoir for *E. coli* in poultry and the features of the bacterial population in intestine has been largely explored in chickens [2]. It is well known that inflammation and necrosis caused by gut pathogens in chickens can substantially affect the structure of gut microbiota. However, such studies are lacking in turkeys. *H. meleagridis* is a well-recognized protozoal parasite that can cause severe caecal tissue destruction in turkeys and the relationship between *H. meleagridis* and *E. coli* is also evident in vitro [34]. Thus, in the present study, *H. meleagridi*s was taken as a model organism to investigate the influence of pathology-associated changes on the caecal microbiota with particular attention to *E. coli.* In order to extend this subject, the ability of *E. coli* to invade the caecal wall was also investigated.

Quantification of the total *E. coli* counts among different groups showed that there was no association between the load of *E. coli* and the pathologies in the gut. The finding is in agreement with a previous study in mice where inflammation and disease severity did not influence the number of *E. coli* [35]. However, a link could be determined between severity of gut inflammation and microbiota composition. The pathology induced by *H. meleagridis* was associated with the pronounced shift in microbial structure, microbial richness and diversity. Similar effects were noticed in chickens that were either co-infected with *H. meleagridis* and avian pathogenic *E. coli* [4] or with *E. tenella* [36, 37]. In controversy, in a different study with *E. tenella* infection in broiler chickens, no changes were reported in alpha diversity even in caecal samples with the highest lesion scores [10]. This discrepancy between studies can be due to different factors such as inoculated pathogen, host, age, stocking density or gender; all have been shown to influence the microbiome composition and diversity [38].

The relative abundance of OTUs belonging to *Anaerotignum lactatifermentans*, *E. coli* and *Faecalibacterium prausnitzii* increased in caecal samples of birds allocated in the severe lesion group. The higher relative abundance of *Anaerotignum lactatifermentans*, amino acid decomposing bacterium, in birds with severe lesions may be attributed to the fibrinous necrotic enteritis [39, 40]. Fibrinous inflammation usually occurs when vasculature fluid leaks allowing large plasma proteins, especially fibrinogen, to enter the tissue [41]. Furthermore, fibrinonecrotic compact mass in caecum blocks the digesta to pass from small intestine and this might result into increased retention time and ultimately flourish the population of proteolytic bacteria [42].

The increase in relative abundance of phylum *Proteobacteria,* in particular *E. coli* population, in birds of the severe lesion group is obviously an indication of gut dysbiosis induced by inflammation and necrosis. However, the high throughput sequencing identifies the overall microbial communities and their relative abundance to each other, in which every decrease in one taxon’s abundance leads to an equivalent increase across the remaining taxa while CFU count determines the absolute bacterial load. Therefore, building up biological interpretations depending only on sequencing data might be misleading as the measurement of each taxon’s relative abundance is dependent on the populations of all other taxa. The enrichment in one bacterial taxa (high relative abundance) does not necessarily reflect the outgrowth of this taxa (actual abundance) [43]. The phenomenon is nicely reflected by the absolute and relative population of *E. coli* in this study. Absolute counts of *E. coli* did not differ across all groups of birds. Relative increase of *E. coli* in birds with severe lesions is therefore a consequence of minimizing other taxa and not *E. coli* overgrowth. Nevertheless, as stated above, it is interesting that *E. coli* maintains the same population in turkeys with or without gut inflammation. Likewise, the relative abundance of *Faecalbacterium prausnitzii*, a potentially beneficial microbe was strongly increased in birds belonging to the severe lesion group. Increase of *Faecalbacterium prausnitzii*, was associated also with an increase of closely related *Fournierella massiliensis* suggesting that the observation was not an artefact. *Faecalibacterium prausnitzii* is an anti-inflammatory commensal bacteria [44] thus, the increased relative abundance of this species in the condition with the highest lesion score might be due to a beneficial response against inflammation [45]. A specific feature of *Faecalibacterium prausnitzii* is the loss of spore formation [46, 47] with alternative measures to cope with oxygen. *Faecalibacterium prausnitzii* is capable to grow at microaerophilic conditions [48] which may enable this bacterium to survive local inflammation associated with generation of oxygen radicals by macrophages and heterophils. Moreover, its ability to produce butyrate and its location in the mucous layer near the epithelial cells [49] might enable it to share an ecological niche with *E. coli* and also supress expansion of pathosymbionts to colonize the mucus layer, however it needs to be further investigated in details.

*Lactobacillus* as a probiotic has been shown to control disease aggravation via modulation of the innate and acquired immune system [50, 51]. Therefore, the high abundance of *Lactobacillus salivarius *and* reuteri* in relatively healthy birds (negative control, no lesion and mild lesion groups) compared to those in severe lesion group with LS 3 or 4 might have contributed to an early immune response, reducing the invasion of *H. meleagridis* and opportunistic pathogenic bacteria along epithelial cells. *Lactobacilli* were observed to be the predominant microbes in the small intestine of chickens and turkeys [52, 53]. Therefore, decreased *Lactobacillus* abundance in birds with typhlitis could also be due to the blockage of digesta from small intestine because of compact fibrino necrotic mass in the caecum. The same explanation can be stated for *Streptococcus* *alactolyticus*, which was also detected with high relative abundance in negative control birds and abundance decreased in infected birds with lesions.

With IHC, *E. coli* could only be detected in luminal content of caecum and preserved caecal content (coagula). Low numbers of *E. coli* positive caecal samples in the actual study can be explained by the fact that routine steps in tissue processing for paraffin sections might remove contents from the gut as previously reported [54]. Anyhow, it is interesting to observe that *E. coli* cells did not infiltrate into the caecal wall even in birds with high relative abundance of *E. coli* and severe sloughing of mucosal epithelium. Similar findings in turkeys from other experimental studies and field cases showed that the pattern remains the same in birds experimentally infected with monoxenic or xenic *H. meleagridis* cultures or in those that are naturally infected. Altogether, it was demonstrated that *E. coli* in the gut of turkeys do not show a tendency to penetrate the gut wall and the effect was not potentiated by the presence of inflammation and necrosis. Very limited re-isolation of *E. coli* from systemic organs with no definite pattern based on severity of lesions indicated absence of systemic translocation of *E. coli* from the gut.

The significant dysbiosis in the caecal microbiota of turkeys was correlated with the severity of pathology following *H. meleagridis* infection. This perturbation was associated with increased relative abundance but not an absolute count of *E. coli* in the gut. Inflammation and necrosis due to *H. meleagridis* did not provoke penetration of caecal wall and systemic translocation of *E. coli* from the gut. Thus, intestinal *E. coli* cells in turkeys do not seem to have a tendency to cause systemic bacteremia even when the gut barrier is compromised.

## Data Availability

Raw sequence reads were uploaded to the NCBI BioProject databank (PRJNA718176).

## Abbreviations

CFU: Colony forming units
Dpi: Days post *H*. *meleagridis* infection
*E.**coli*: *Escherichia* *coli*
*H*. *meleagridis*: *Histomonas* *meleagridis*
*E. tenella*: *Eimeria tenella*
IHC: Immunohistochemistry
LS: Lesion score
OTU: Operational taxonomic unit
PBS: Phosphate buffered saline
PCR: Polymerase chain reaction
